# Facial nerve neurographies in intensive care unit-acquired weakness

**DOI:** 10.1186/s42466-023-00275-3

**Published:** 2023-09-21

**Authors:** Maximilian Lochter, Martin Sommer, Onnen Moerer, Caspar Stephani

**Affiliations:** 1 Department of Anesthesiology, University Medical Center Göttingen, Robert-Koch-Straße 40, 37075 Göttingen, Germany; 2 Department of Urology, University Medical Center Göttingen, Robert Koch-Str. 40, 37075 Göttingen, Germany; 3 Department of Neurology, University Medical Center Göttingen, Robert Koch-Str. 40, 37075 Göttingen, Germany

**Keywords:** Nerve conduction studies, Critical illness, Intensive care unit acquired weakness, Orbicularis oculi reflex, Facial nerve

## Abstract

**Background:**

Patients with an intensive care unit-acquired weakness (ICU-AW) often present clinically with severe paresis of the limb and trunk muscles while facial muscles appear less affected. To investigate whether the facial nerves are partially spared from this condition, we performed both peripheral and cranial nerve conduction studies in critically ill patients.

**Methods:**

In patients requiring prolonged ICU therapy, the motor and sensory nerve conduction velocities of the peroneal, ulnar and facial nerves and the muscle action potentials of the associated muscles, as well as the orbicularis oculi reflexes were assessed shortly after admission, and on ICU days 7 and 14.

**Results:**

Eighteen patients were included in the final data analysis (average age 54.2 ± 16.8 years, 8 females). The amplitudes of the peroneal nerve compound muscle action potentials (CMAPs) were reduced in all patients at ICU days 7 and 14 (F(1.39; 23.63) = 13.85; *p* < 0.001). There was no similar decrease in the CMAP amplitudes of the ulnar or facial nerve. Other parameters of nerve function (latencies, sensory and motor nerve conduction velocities, sensory nerve action potentials) remained unchanged. The reproducibility of the orbicularis oculi reflex was reduced during the disease course, while its latencies did not change significantly during the disease course.

**Conclusions:**

There is a relative preservation of CMAPs in facial and hand as opposed to foot muscles. This is compatible with the clinical observation that the facial muscles in patients with ICU-AW are less severely affected. This may be primarily a function of the nerve length, and consequently more robust trophic factors in shorter nerves.

*Trial registration* This study was prospectively registered in the German Clinical Trial Register on April 20th 2020 (DRKS00021467).

**Supplementary Information:**

The online version contains supplementary material available at 10.1186/s42466-023-00275-3.

## Background

The intensive care unit acquired weakness (ICU-AW) is a frequent and prognostically relevant complication of critically ill patients. Its predominating cause may be either a critical illness neuropathy—affecting more distal muscles—or a critical illness myopathy—affecting more proximal muscles. Frequently, however, a combination of both processes is found [8]. The main symptom of this disorder is a flaccid, painless weakness affecting both distal and proximal parts of the extremities, as well as the trunk in more severe cases. Aside of the typical clinical manifestation, nerve conduction studies are the most reliable method for diagnosing this disorder [8]. It is recommended to use conduction studies of the peroneal and one sensory (primarily the sural) nerve to screen patients with suspected ICU-AW, while bearing in mind, that this diagnostic set-up alone generally is not suitable to safely distinguish between critical illness neuro- and myopathy [9, 12, 16]. Clinically, a discrepancy between mostly spared facial and the more severely affected peripheral muscles can often be observed [8]. However, we are not aware of any systematic electrophysiological studies of possible nerve conduction differences between facial and peripheral nerves in patients afflicted with ICU-AW. We therefore performed electrophysiological studies on the peroneal, ulnar and facial nerves measuring nerve conduction velocities and eyelid reflexes. We assessed to which degree facial nerves are affected in patients with critical illness and ICU-AW.

## Methods

After approval by the local ethics committee of the University of Göttingen (No. 21/8/19), in accordance with the Declaration of Helsinki, study inclusion was performed after written informed consent of the patient or his legal representative.

### Study cohort

Adult patients (≥ 18 years of age) who were considered on admission likely to remain in the ICU for at least seven days were recruited. We excluded patients who had to be isolated due to colonization with multidrug-resistant bacteria or an infectious disease such as COVID-19. Likewise, patients with a known polyneuropathy, a Guillain-Barré-syndrome, myasthenia gravis, known facial palsy, and patients after resuscitation or in acute delirium were not considered for this study.

### Study design

We performed a prospective observational diagnostic trial. Measurements took place at three time points after admission to the ICU: (1) within 48 h, (2) at day 7(± 1), and (3) at day 14 (± 1) after admission. At each time point the electrophysiological measurements were performed in the following sequence: motor neurography—right peroneal and right ulnar nerve (the latter together with sensory neurography), followed by bilateral orbicularis oculi reflex and bilateral facial nerve neurography.

We collected demographic as well as clinical data, which are established or potential risk factors for developing ICU-AW: age, height, blood sugar levels, edema (yes or no), sepsis (according to the list of diagnoses in the patients’ electronic medical records), administration of hydrocortisone (in mg per patient and day) or neuromuscular blockers (in mg per patient), administered sedatives (type and average dose per day), numerical values of the Richmond Agitation-Sedation Scale (RASS) and the Simplified Acute Physiology Score II (SAPS II). A clinical and neurological status assessment including evaluation of motor strength using the Medical Research Council scale (MRC-scale) was performed at each visit.

### Study procedures

The patients were examined in their beds in the ICU with a mobile electrodiagnostic system (hardware Nicolet AT2 + b Amplifier, software Viking®, Natus®, Orlando, USA). After proper positioning of the extremity, the skin was cleaned (including Coloplast®), and the electrodes (either reusable or single-use Red Dot 2239 ECG-electrodes, 3 M®, Saint Paul, Minnesota, USA) were attached with the reference electrode located 2–3 cm from the active electrode above the muscle belly and a ground electrode in between. Stimulation started using a pulse duration of 0.1 ms at low intensity (e.g., 5 mA), which we incrementally increased in 5 mA steps until reaching a supramaximal response. In the presence of relevant edema, the pulse duration was increased from 0.1 to 0.2 or 0.5 ms.

For studying the peroneal nerve, the active electrode was placed on the belly of the extensor digitorum brevis muscle. Distal stimulation was above the proximal dorsal foot close to the tendon of the anterior tibial muscle, and proximal stimulation was at the caput fibulae. Sensory and motor conduction studies of the ulnar nerve were performed simultaneously, with the active electrode attached above the abductor digiti minimi muscle belly, and ring electrodes on the fifth finger. Distal stimulation was applied on the ulnar side of the distal forearm 7 cm from the electrode on the abductor digiti minimi muscle with proximal stimulation at the sulcus nervi ulnaris.

For electrophysiological measurements of the orbicularis oculi reflex, the active electrodes were placed bilaterally under the eye above the lower part of the orbicularis oculi muscle. The indifferent and the ground electrode were placed above the glabella. We stimulated the supraorbital nerve on each side sequentially and separately averaged the results of the five measurements on each side. We recorded the latencies of the ipsilateral direct R1 response, as well as those of ipsilateral and contralateral indirect R2-responses.

To study the motor nerve conduction of the facial nerve, we kept the electrodes used for measuring the orbicularis oculi reflex in place and stimulated directly in front of the ear.

To calculate the nerve conduction velocities for each nerve conduction study, we measured the distances between electrodes and stimulation points, as well as between two stimulation points (not applicable for the facial nerve).

### Statistics

Descriptive statistics were provided by calculating means and standard deviations for age, height, disease severity scores. For the final analysis of our nerve conduction studies we included amplitude of the CMAP (measured baseline to peak), duration of CMAP, latency of the CMAP as well as nerve conduction velocity (not applicable to the facial nerve). Applying the Mauchly test, we tested for sphericity of our data, which was accepted when the test statistics showed a level of significance > 0.05. In other cases, we applied a correction according to Greenhouse–Geisser, to avoid a type I error. The effect of edema on CMAP amplitude was investigated by using a one-tailed t test.

For the final statistical analysis, we applied a general linear model for repeated measures, the dependent variable being the amplitude of either CMAPs or SNAPs and the inner-subject factor being time. The level of significance was set at 0.05. For post hoc testing we applied a Bonferroni correction to avoid alpha error inflation. We used the software Excel^®^ (Microsoft Office) as well as SPSS 28^®^ (IBM).

## Results

We included 24 patients between July 2020 and March 2022. Three patients died during the study period from their severe critical illness and completely unrelated to the nerve conduction studies of this investigation, two patients or their legal guardian retracted their consent, and one patient was discharged prior to the last measurement. Hence, complete measurements were available for 18 patients (54.17 ± 16.81 (21–74) years of age; height: 174 ± 9 cm; 8 females), whose data we subjected to the final data analysis. Of these, seven had sustained a severe subarachnoid hemorrhage, four had multiple severe injuries, three an acute respiratory distress syndrome (ARDS), two an intracerebral hemorrhage, and two a large vessel occlusion stroke.

According to the RASS scores the patients recovered only moderately from high levels of inactivity and/or sedation during the study from − 4.77 ± 0.43 on day one, to − 4.28 ± 0.75 on day seven, and to − 3.11 ± 1.71 on day 14. The average SAPS II scores as correlates of overall disease severity remained at essentially the same level with 33.4 ± 9.15 points on day one, 37.67 ± 10.52 on day seven, and 32.33 ± 9.65 on day 14. Unfortunately, it was not possible to reliably examine the muscle strength (i.e., full MRC scale) due to the severity of illness (e.g. coma), a lack of cooperation and/or the severe flaccid paresis in all patients. Data on clinical examination, mechanical ventilation and levels of sedation on day 14 of the study are provided with Additional file 1.

**Fig. 1:**
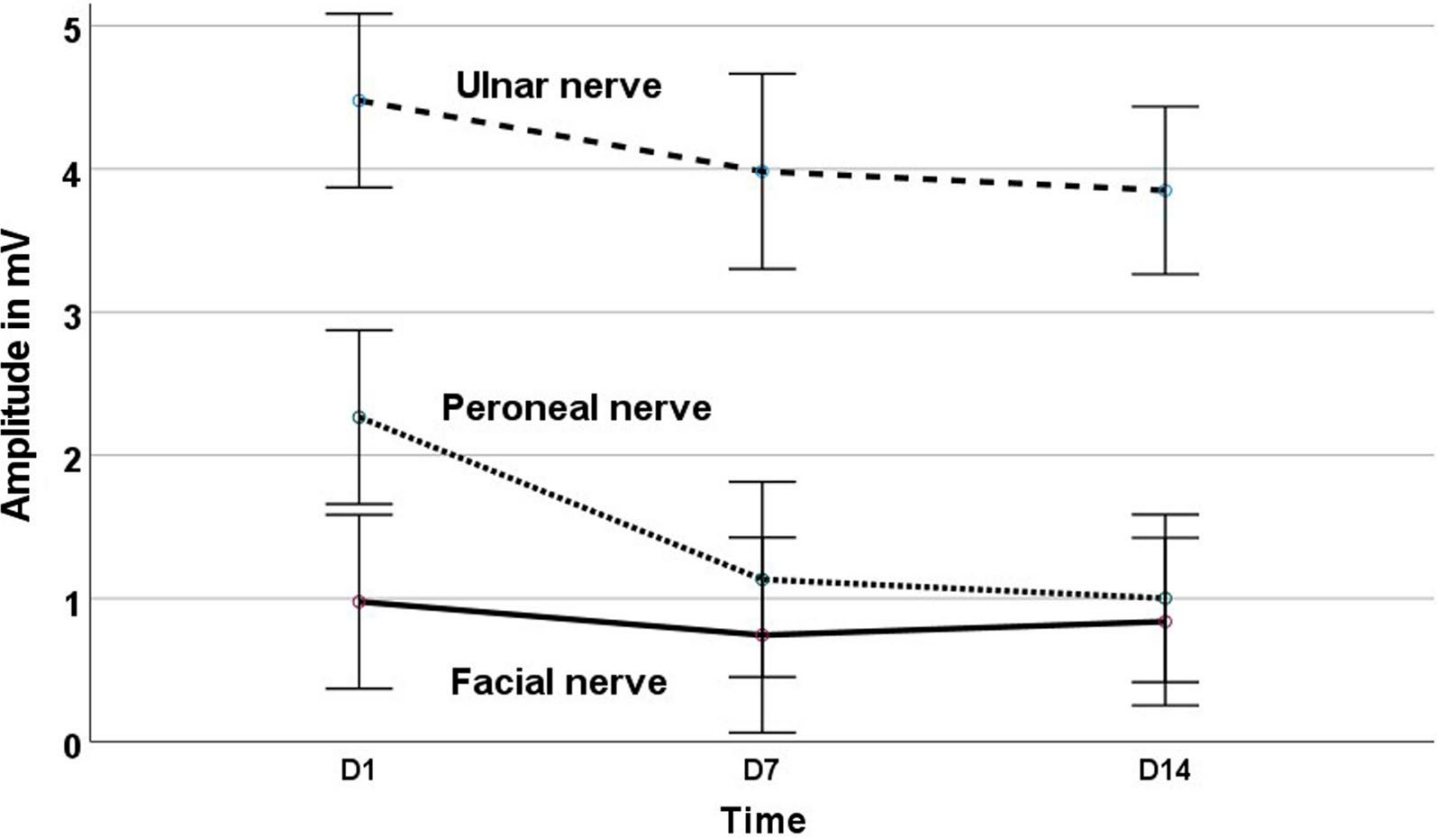
Line graphs demonstrating the amplitudes of the compound muscle action potentials in mV (y-axis) of ulnar, peroneal, and facial nerve neurographies within the course of the study. D1, D7, D14 = days 1, 7, and 14. Error bars indicate the 95% confidence intervals. Detailed line graphs with the course of the amplitudes for each single patient can be found in Additional file 2

**Fig. 2:**
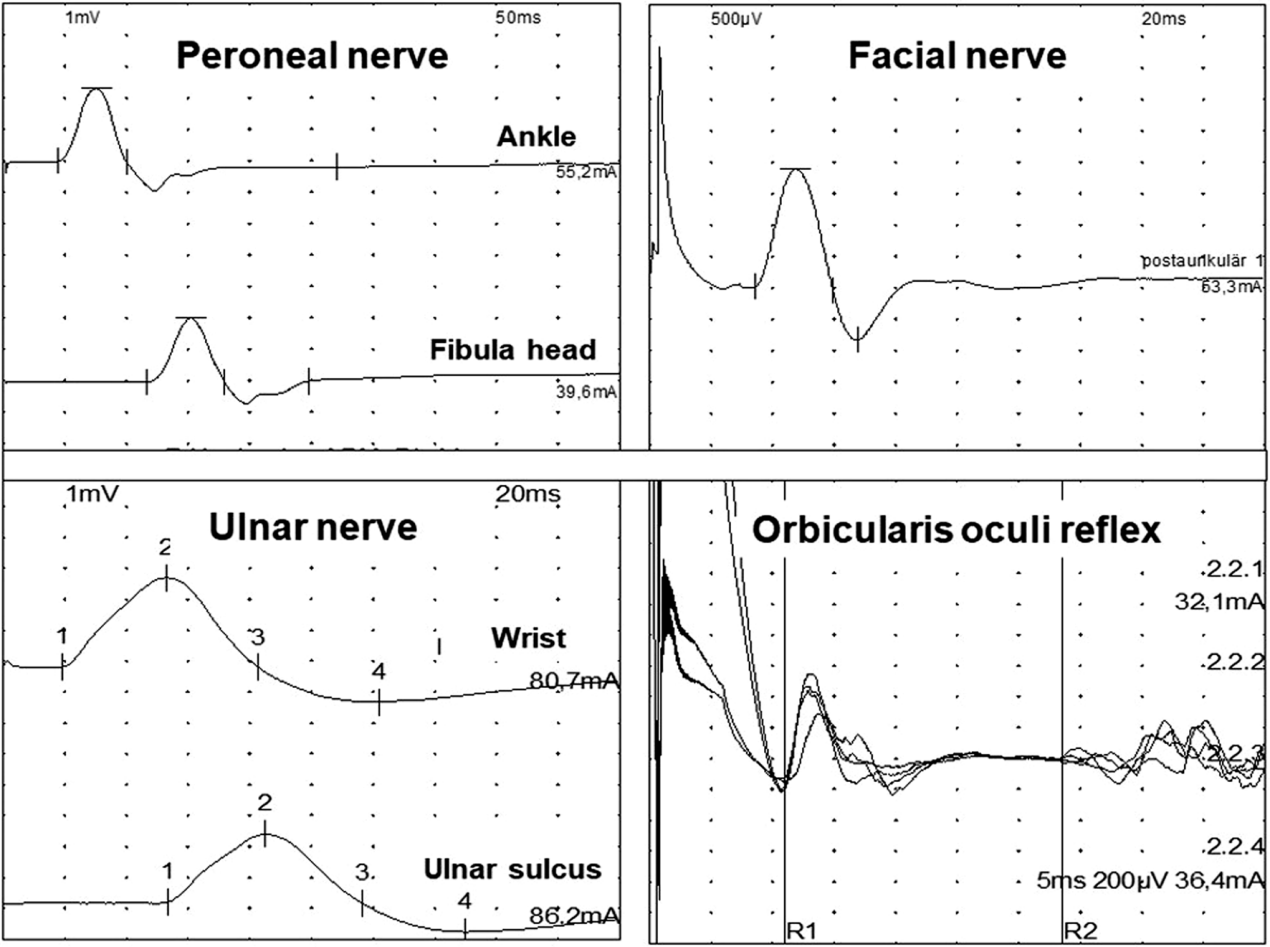
Selected original tracings of the motor conduction velocities and the orbicularis oculi reflex measurements conducted in this study. Please note the different scales

Over the duration of the study, the amplitude of the CMAP after stimulation of the right peroneal nerve diminished significantly, both with distal (2.26 ± 1.37 mA vs. 1.13 ± 0.96 mA vs. 1 ± 0.93 mA; *p* < 0.001; n = 18), and with proximal stimulation (1.89 ± 1.27 mA vs. 0.99 ± 0.83 mA vs. 0.9 ± 0.86 mA; *p* = 0.002; n = 17) (Additional file 2 for all CMAPs after distal stimulation of the peroneal nerve). We were unable to evoke CMAPs after proximal stimulation at the fibular head in one patient, hence stimulating in the popliteal fossa instead (for days 7 and 14). Post hoc testing revealed significant differences for the two stimulation sites between the first and second measurements (*p* = 0.002 and *p* = 0.004, respectively) as well as between the first and third (*p* = 0.04 and *p* = 0.02, respectively). The findings were similar with respect to area of the CMAPs after distal (10.72 ± 7.85 cm^2^ vs. 5.62 ± 4.53 cm^2^ vs. 5.51 ± 4.62 cm^2^; *p* = 0.004) and proximal stimulation (8.5 ± 7.03 cm^2^ vs. 4.93 ± 3.83 cm^2^ vs. 4.75 ± 3.87 cm^2^; *p* = 0.01; n = 17); with significant differences between first and second (*p* = 0.01 distal and proximal) as well as first and third measurements (*p* = 0.03 distal and proximal). By contrast, CMAP amplitudes after stimulation of the right ulnar nerve did not change significantly during the study duration (4.48 ± 1.62 mA vs. 3.98 ± 2.21 mA vs. 3.85 ± 1.82 mA; *p* = 0.36; n = 18 at wrist; 3.78 ± 1.82 mA vs. 3.51 ± 2.15 mA vs. 3.4 ± 1.93 mA; *p* = 0.72; n = 18 at sulcus ulnaris) (Additional file 2). Likewise, the recordings after facial nerve stimulation were essentially unchanged, both for amplitudes and for the area under the curve (left side amplitude: 0.93 ± 0.71 mA vs. 0.77 ± 0.57 mA vs. 0.82 ± 0.69 mA; *p* = 0.48; left side area: 2.77 ± 2.05 cm^2^ vs. 3.1 ± 2.62 cm^2^ vs. 2.66 ± 1.93 cm^2^; *p* = 0.68; n = 18; and right side amplitude: 0.98 ± 0.64 mA vs. 0.74 ± 0.66 mA vs. 0.84 ± 0.59 mA; *p* = 0.24; right side area: 2.97 ± 2.27 cm^2^ vs. 2.24 ± 1.53 cm^2^ vs. 2.88 ± 2.24 cm^2^; *p* = 0.25 n = 18); Interindividual variability was high after stimulation of the facial and ulnar nerves (Fig. 1, Table 1). Representative motor nerve conduction velocity traces are shown in Fig. 2.

**Table 1:** Amplitudes of the compound motor action potentials after nerve conduction studies of four nerves in mV ± standard deviation at baseline (day 1)

	Amplitude day 1	-day 7/day 1	-day 14/day 1
*Amplitude (mV)*			
Peroneal nerve			
Ankle	2.19 ± 1.38	0.45 (0.98 ± 0.71)	0.42 (0.93 ± 0.96)
Head of fibula	1.8 ± 1.25 (n = 17)	0.48 (0.87 ± 0.66)	0.46 (0.83 ± 0.83)
Ulnar nerve			
Wrist	4.48 ± 1.62	0.89 (3.98 ± 2.21)	0.86 (3.85 ± 1.82)
Ulnar sulcus	3.78 ± 1.82	0.93 (3.51 ± 2.15)	0.9 (3.42 ± 1.93)
Left facial nerve	0.93 ± 0.71	0.83 (0.77 ± 0.57)	0.88 (0.82 ± 0.7)
Right facial nerve	0.98 ± 0.64	0.76 (0.74 ± 0.66)	0.86 (0.84 ± 0.59)

For day 1 CMAP amplitudes are given in mV ± standard deviation. For days 7 and 14 the first value displays the ratio of amplitudes of the respective day compared to the baseline measurement with the actual amplitudes in mV and standard deviations in brackets (patient per patient results are given in Additional file 2)

The duration of the CMAP remained stable after stimulation of the peroneal nerve (distal (6.11 ± 1.64 ms vs. 5.94 ± 1.47 ms vs. 6 ± 1.34 ms; *p* = 0.89); proximal (6.3 ± 2.2 ms vs. 6.78 ± 1.59 ms vs. 6.79 ± 1.61 ms; *p* = 0.92; n = 17)), the ulnar nerve (distal (5.65 ± 1.01 ms vs. 5.6 ± 0.86 ms vs. 5.72 ± 1.02 ms; *p* = 0.93); proximal (6.06 ± 0.85 ms vs. 6.11 ± 1.07 ms vs. 6.08 ± 1.23 ms; *p* = 0.99)), and the left (5.23 ± 1.88 ms vs. 5.75 ± 1.9 ms vs. 5.53 ± 1.26 ms; *p* = 0.41) as well as the right facial nerves (5.01 ± 1.66 ms vs. 5.35 ± 2.11 ms vs. 6.01 ± 2.21 ms; *p* = 0.27).

In addition, there were no significant changes in the motor nerve conduction velocities for the peroneal nerve (45 ± 6 m/s vs. 46 ± 4 m/s vs. 46 ± 4 m/s; *p* = 0.57; n = 17) as well as the ulnar nerve (59 ± 7 m/s vs. 59 ± 9 m/s vs. 59 ± 5 m/s; *p* = 0.85), nor did latencies of the CMAPs after stimulation of the left (3 ± 0.0.68 ms vs. 2.61 ± 0.47 ms vs. 3.01 ± 0.85 ms; *p* = 0.08) and right facial nerve (2.7 ± 0.71 ms vs. 2.43 ± 0.69 ms vs. 2.63 ± 0.57 ms; *p* = 0.29) change during the study (Table 2).

**Table 2:** Average nerve conduction velocities (NCV) of the nerves examined in this study

	NCV day 1	Day 7/day 1	Day 14/day 1
*Nerve conduction velocity (m/s) and latencies (ms), respectively*			
Peroneal nerve conduction velocity	44.88 ± 5.58 (n = 17)	1.03 (46.12 ± 4.01)	1 (44.79 ± 4.87)
Ulnar motor nerve conduction velocity	59.13 ± 6.63	1 (59.4 ± 8.68)	0.99 (58.52 ± 5.38)
Left facial nerve CMAP-latency	3 ± 0.68	0.87 (2.61 ± 0.47)	1 (3.02 ± 0.85)
Right facial nerve CMAP-latency	2.69 ± 0.71	0.9 (2.42 ± 0.69)	0.98 (2.63 ± 0.57)

For day 1 CMAP amplitudes are given in mV ± standard deviation. For days 7 and 14 the first value displays the ratio of nerve conduction velocities of the respective day compared to the baseline measurement with the actual velocity in ms and standard deviations in brackets (patient per patient results are given in Additional file 2)

We additionally performed an electrophysiological study of one sensory ulnar nerve and calculated the negative peak (NP) amplitude (maximum negative deflection of the sensory nerve action potential from the baseline). Neither after distal stimulation at the wrist (17.1 ± 13.08 mA vs. 13.7 ± 7.76 mA vs. 15.2 ± 9.04 mA; *p* = 0.76; n = 10) nor after proximal stimulation at the sulcus ulnaris (17.3 ± 16.66 mA vs. 4.33 ± 3.14 mA vs. 5.67 ± 2.16 mA; *p* = 0.12; n = 6) did we observe significant differences in the amplitudes during the course of the investigation. However, the intraindividual reproducibility of SNAPs was poor allowing only nine and six full consecutive measurements after distal and proximal stimulation, respectively (Table 3).

**Table 3:** Sensory nerve action potential (SNAP) amplitudes during the course of the investigation

	SNAP at day 1	Day 7/day 1	Day 14/day 1
*NP amplitude (μV)*			
Ulnar nerve			
Wrist	14.36 ± 12.24 (n = 14)	0.97 (14 ± 7.08 (n = 12))	1.1 (15.86 ± 9.77 (n = 14))
Ulnar sulcus	13.75 ± 15.57 (n = 8)	0.29 (3.99 ± 3.26 (n = 11))	0.4 (5.45 ± 3.47 (n = 11))

For day 1 SNAP amplitudes are given in µV ± standard deviation at baseline. For days 7 and 14 the first value displays the ratio of amplitudes of the SNAPs of the respective day compared to the baseline measurement with the actual velocity in ms and standard deviations in brackets (patient per patient results are given in Additional file 2)

After left-sided stimulation the orbicularis oculi reflex was evocable in half of the patients at days 1 and 7, and in 13 patients at day 14. After right-sided stimulation it was evocable in 11, 10, and 12 patients on days 1, 7, and 14, respectively). Latencies of the ipsilateral R1-response on the left side (11.43 ± 0.67 ms vs. 11.73 ± 1.27 ms vs. 11.72 ± 0.93 ms; *p* = 0.8; n = 6) as well as on the right side (11.19 ± 0.68 ms vs. 11.69 ± 1.24 ms vs. 11.54 ± 0.81 ms; *p* = 0.37; n = 8) remained stable throughout the observation period (Table 4). The reliability of R2-responses was poor, with up to 3 responses at the first measurement and up to 7 responses at the third measurement depending on side of stimulation and direct or indirect responses.

**Table 4:** Latency of the ipsilateral R1 response of the orbicularis oculi reflex in ms

	Latency at day 1	Day 7/day 1	Day 14/day 1
*R1-response ipsilateral (ms)*			
Left	11.79 ± 1.23 (n = 9)	0.99 (11.69 ± 1.07 (n = 9))	1.01 (11.9 ± 1.48 (n = 13))
Right	11.34 ± 1.41 (n = 11)	1.03 (11.73 ± 1.11 (n = 10))	1.01 (11.4 ± 0.94 (n = 12))
Left–right	0.73 ± 0.79 (n = 9)	1 (0.73 ± 0.25 (n = 8))	0.96 (0.7 ± 0.72 (n = 9))

For day 1 SNAP amplitudes are given in µV ± standard deviation at baseline. For days 7 and 14 the first value displays the ratio of amplitudes of the SNAPs of the respective day compared to the baseline measurement with the actual velocity in ms and standard deviations in brackets (patient per patient results are given in Additional file 2)

With regard to the typical risk factors often associated with the occurrence of ICU-AW, blood sugar concentrations remained stable at mildly hyperglycemic levels (144 ± 33 mg/dl (day 1), 139 ± 26 mg/dl (day 7), 141 ± 24 mg/dl (day 14)), three patients received 200 mg hydrocortisone over 24 h via continuous infusion, four patients were diagnosed with sepsis, and no patient received a total dose of the neuromuscular blocker rocuronium exceeding 100 mg. Due to the low prevalence of these factors, we refrained from a formal statistical analysis on the effects of these factors on nerve conduction.

## Discussion

This study in critically ill patients showed that the CMAP amplitudes elicited by stimulating the peroneal nerve differ from those observed after stimulation of the ulnar and facial nerves. While the stimulated peroneal CMAPs decreased significantly in nearly all patients after one week of critical illness, those observed after stimulation of the ulnar and facial nerves did not change significantly. The conduction velocities of the nerves under study remained stable during the study period, as were the latencies of the orbicularis oculi reflex, although this latter was only evocable in about half of the patients. These results are at least compatible with our hypothesis that the facial nerves are less severely affected in ICU-AW as described in the review of Hermans and Van den Berghe [8]. However, a prominent involvement of the facial nerves has been reported in some studies [4, 7].

### Prevalence

Reduced CMAP amplitudes with unchanged nerve conduction velocities are a pattern typical for ICU-AW [18], and we detected this combination in all but one patient of our cohort. However, six of these patients were unresponsive at the last visit (day 14) due to deep sedation or persistent coma. Hence, we were not able to make a final diagnosis on clinical grounds in this one third of our cohort, while we saw both, reduced CMAP-amplitudes after peroneal nerve stimulation and severe flaccid pareses, in roughly 60% of our cohort. This 60% prevalence exceeds the 40% typically described in the literature [19]. This difference is possibly due to a positive selection for disease-severity in our cohort, which included only patients considered on admission to have a high likelihood of requiring intensive care for a prolonged period (i.e., patients with severe subarachnoid hemorrhage, intracranial bleeding, multiple traumatic injuries). Hence, this reminds to take into consideration the cohort or population when reporting the prevalence of ICU-AW acknowledging that in other studies its prevalence may have been reported in ICU-patients less preselected for disease severity [11].

### Distribution

The more pronounced manifestation in the lower extremities as seen in this study is a common finding [6] and may be related to nerve length. The longer a nerve, the longer regenerative processes will take, since transport mechanisms start from the cell body which serves as main source of protein synthesis in a cell [6, 15]. In addition, the microcirculation in peripheral nerves may be impaired more strongly and at an earlier time in critically ill patients leading to accumulation of toxic metabolites and predisposing to ischemic injuries [12]. Moreover, increased length, surface and volume of a nerve may increase the sheer likelihood of being injured and dysfunctional. Hence, shorter nerves, such as the facial nerve, may be less vulnerable in such circumstances. On the other hand, physiological differences between muscles may contribute to different responses to critical illness. In animal models, the muscles of the extremities show a decreased expression of protective heat shock proteins in models of critical illness, as compared to facial muscles [6]. However, for technical reasons, there is a clear bias towards measuring from distal muscles and thus registering the function of longer nerves. Thus, more proximal nerves may show prominent electrophysiological alterations as well. In fact, in order to gain a better picture of time course and pattern of distribution of ICU-AW, a further study may compare other more special neurographies (e.g. femoral nerve neurography, Hoffmann’s reflex) with those commonly obtained. Surprisingly, CMAP-amplitudes of the rather long ulnar nerves did not change significantly in our cohort. While there was a reduction of at least 30% of CMAP amplitudes after stimulation of the ulnar nerve in half of the patients, there was stability or even increase in CMAP amplitudes at day 7 in the other half indicating a more variable course of changes (Additional file 2).

### Time course

CMAP amplitudes after peroneal nerve stimulation were already reduced at the first measurement compared to external reference values (e.g., 5.1 ± 2.3/2 mV) [5], but also to our in-house reference values. They decreased further thereafter, while CMAP amplitudes after stimulation of the ulnar and facial nerves were mostly normal or nearly normal at study onset and did not change significantly during the study period [5, 17]. The lower CMAP amplitudes after peroneal nerve stimulation may indicate either undiagnosed preexisting neuropathies in some of the patients, or a rapid development of neuromyopathic changes during the first 48 h of critical illness. The changes in the CMAP amplitudes after stimulation of the peroneal nerve generally occurred within the first week of critical illness and remained stable thereafter. Therefore, early processes are apparently decisive in the induction of changes leading to an ICU-AW. In the second week of critical illness degenerative and regenerative mechanisms were possibly already balanced in this group of patients. Early hypoexcitability of the membranes of muscles of the extremities, due to changes in membrane channels contribute to this dynamic [6]. Likewise, immobility contributes to proteolysis, which is most intense in early disease stages [14]. In addition, mechanical ventilation, regarded as a risk factor for developing an ICU-AW, is more common during early stages [13]. Moreover, hypoxemia in patients suffering from ARDS or sepsis is often most severe in early acute stages and may contribute to a reduced oxygen supply in peripheral nerves and lead to development of neurogenic edema [1]. However, the exact mechanisms underlying the typical time course of induction of and regeneration from nerve and muscle injuries of patients with ICU-AW are hitherto incompletely understood [2, 3, 6].

### Limitations

Unfortunately, several factors, such as severity of the illness or inability of the patient to cooperate due to deep sedation of muscle relaxation, prevented us from directly assessing the muscle strength, and contrary to the study design, a valid electroclinical correlation was not possible. Similarly, we did not correlate the electrophysiological findings with typical risk factors such as use of glucocorticoids, neuromuscular blockers, diagnosis of sepsis or hyperglycemia being too low in number. In addition, since we did not perform electromyography or direct muscle stimulation in this study, we are unable to differentiate to which degree a neuro- or myopathy was the leading cause of the ICU-AW. The sensory nerve action potentials of the ulnar nerve did not change significantly during the two-week study period, which would have been an indirect indicator of the contribution of a myopathy to the reduced CMAPs. However, the reproducibility of the SNAPs was reduced, possibly due to a greater liability for artifacts as well as edema-associated signal reduction [10] thus affecting the quality of this measurement. Generally, measurements of the SNAPs help to differentiate neuropathic from myopathic processes. However, this study was underpowered to compensate for missing data and high variability of sensory nerve action potentials. On the other hand, the duration of the CMAPs after stimulation of the peroneal nerve also remained stable. With myopathy, one often observes an increase in the duration of the CMAP in addition to a decrease in amplitude. In fact, the average CMAP amplitude duration did not increase in our cohort, which argues against the diagnosis of a predominant critical illness myopathy. And the fact, that CMAP amplitudes of the extensor digitorum brevis muscle—representing a most distal location—decreased substantially, indicates existence of a neuropathy. However, this study was not designed to differentiate between the two pathophysiological elements possibly contributing to ICU-AW. Additionally, it may be not surprising that we did not find significant changes during the course of the critical illness by measuring the orbicularis oculi reflex given that we only can reliably compare latencies in the case of this reflex. However, latencies or nerve conduction velocities commonly remain hardly affected by critical illness neuropathies.

## Conclusions

In summary, we found reduced muscle action potential amplitudes in the lower extremity, but not in the upper extremity or the face during the first two weeks after ICU admission in a small sample of critically ill patients. This is in agreement with clinical observations, which often highlight the preserved facial motor activity in critically ill patients, who otherwise suffer from severe tetraparesis.

### Supplementary Information

**Additional file 1.** Clinical observation and examination on day 14. Patient characteristics: Age, diagnosis, sedation level, motor activity, ocular motility, cranial nerve status, mechanical ventilation, weakness.

**Additional file 2.** CMAPs in mV after distal stimulation of the peroneal, ulnar and right facial nerve and SNAPs after distal stimulation of the ulnar nerve. Course of the CMAPs after distal stimulation of the peroneal, ulnar and right facial nerve and course of the SNAPs after distal stimulation of the ulnar nerve during the study for each patient included, presented graphically as well as in a table.

## Data Availability

The datasets used and/or analysed during the current study are available from the corresponding author on reasonable request.

## Abbreviations

CMAP: Compound motor action potential
ICU: Intensive care unit
ICU-AW: Intensive Care Unit aquired weakness
MRC: Medical Research Counsil
NP: Negative peak
RASS: Richmond Agitation and Sedation Scale
SNAP: Sensory nerve action potential

## References

[1] Batt J, dos Santos CC, Cameron JI, Herridge MS (2013). Intensive care unit–acquired weakness: Clinical phenotypes and molecular mechanisms. American Journal of Respiratory Critical Care Medicine.

[2] Batt J, Herridge MS, Dos Santos CC (2019). From skeletal muscle weakness to functional outcomes following critical illness: A translational biology perspective. Thorax.

[3] Bloch S, Polkey MI, Griffiths M, Kemp P (2012). Molecular mechanisms of intensive care unit-acquired weakness. European Respiratory Journal.

[4] Borré-Naranjo D, Rodríguez-Yánez T, Almanza-Hurtado A, Martínez-Ávila MC, Dueñas-Castell C (2022). Intensive care unit acquired weakness associated with acute severe asthma. Clinical Medicine Insights: Case Reports.

[5] Dyck PJ, Thomas PK (2005). Peripheral neuropathy.

[6] Friedrich O, Reid MB, Van den Berghe G, Vanhorebeek I, Hermans G, Rich MM, Larsson L (2015). The sick and the weak: Neuropathies/myopathies in the critically ill. Physiological Reviews.

[7] Gurjar M, Azim A, Baronia AK, Poddar B (2010). Facial nerve involvement in critical illness polyneuropathy. Indian Journal of Anaesthesia.

[8] Hermans G, Van den Berghe G (2015). Clinical review: Intensive care unit acquired weakness. Critical Care.

[9] Kelmenson DA, Quan D, Moss M (2018). What is the diagnostic accuracy of single nerve conduction studies and muscle ultrasound to identify critical illness polyneuromyopathy: A prospective cohort study. Critical Care.

[10] Lacomis D (2013). Electrophysiology of neuromuscular disorders in critical illness. Muscle and Nerve.

[11] Latronico N, Bolton CF (2011). Critical illness polyneuropathy and myopathy: A major cause of muscle weakness and paralysis. Lancet Neurology.

[12] Latronico N, Nattino G, Guarneri B, Fagoni N, Amantini A, Bertolini G, GiVITI Study Investigators (2014). Validation of the peroneal nerve test to diagnose critical illness polyneuropathy and myopathy in the intensive care unit: The multicentre Italian CRIMYNE-2 diagnostic accuracy study. F1000Research.

[13] Nanas S, Kritikos K, Angelopoulos E, Siafaka A, Tsikriki S, Poriazi M, Kanaloupiti D, Kontogeorgi M, Pratikaki M, Zervakis D, Routsi C, Roussos C (2018). Predisposing factors for critical illness polyneuromyopathy in a multidisciplinary intensive care unit. Acta Neurologica Scandinavica.

[14] Patel BK, Pohlman AS, Hall JB, Kress JP (2014). Impact of early mobilization on glycemic control and ICU-acquired weakness in critically ill patients who are mechanically ventilated. Chest.

[15] Said G (2007). Neuropathy: A review. Nature Clinical Practice Neurology.

[16] Stevens RD, Marshall SA, Cornblath DR, Hoke A, Needham DM, de Jonghe B, Ali NA, Sharshar T (2009). A framework for diagnosing and classifying intensive care unit-acquired weakness. Critical Care Medicine.

[17] Stöhr M, Pfister R (2014). Klinische Elektromyographie und Neurographie – Lehrbuch und Atlas.

[18] Tankisi H, de Carvalho M, Z’Graggen WJ (2020). Critical illness neuropathy. Journal of Clinical Neurophysiology.

[19] Vanhorebeek I, Latronico N, Van den Berghe G (2020). ICU-acquired weakness. Intensive Care Medicine.

