# Supplementary material for: Facial nerve neurographies in intensive care unit-acquired weakness
Source: Neurol Res Pract. 2023 Sep 21;5:50. doi: 10.1186/s42466-023-00275-3 (PMC10512492; doi:10.1186/s42466-023-00275-3)
Supplement: Supplementary file 1 — Additional file 1. Clinical observation and examination on day 14. Patient characteristics: Age, diagnosis, sedation level, motor activity, ocular motility, cranial nerve status, mechanical ventilation, weakness. [file 42466_2023_275_MOESM1_ESM.docx]

# Additional file 1

# Clinical observation and examination on day 14

|  | **Age** | **Diagnosis leading to hospital admission** | **Sedation levels on day 14** | **Motor activity** | **Ocular motility** | **Cranial nerve status** | **Mechanical ventilation** | **ICU-AW**  **Clinical** |
| --- | --- | --- | --- | --- | --- | --- | --- | --- |
| P1 | 50 | Acute respiratory distress syndrome | Sufentanil: 36 µg/h  Dexmedetomidine: 54 µg/h | None | none | No signs of activity | Y | - |
| P2 | 41 | Subarachnoid hemorrhage with right hemispheric infarction | none | Moves all extremities, MRC 3/5 in right hand | Pupils equal round reactive to light and accommodation (PERRLA) | No pathological findings | N | Y |
| P3 | 33 | Polytrauma | Sufentanil 11,7 µg/h  Dexmedetomidine: 182 µg/h | Spontaneous movement of the right arm, no movement of other extremities | PERRLA | Moves head into the direction of sound | Y | Y |
| P4 | 61 | Polytrauma | Sufentanil: 25 µg/h  Midazolam 3,6 mg/h | none | Pupils isocor, delayed light reaction | No signs of activity | Y | - |
| P5 | 73 | Middle cerebral artery infarction on the right | none | Spontaneous movement of the right upper and lower extremities; does not obey commands, moves to localize pain | PERRLA | Eyes mostly closed | N | N |
| P6 | 78 | Polytrauma | none | Spontaneous movement of the left arm, movement of the legs (left > right) on painful stimulus; arm-stressed hemiparesis on the right side | PERRLA | No pathological findings | N | Y |
| P7 | 67 | Middle cerebral artery infarction | none | none | Pupils isocor, not reactive to light | No signs of activity | Y | - |
| P8 | 21 | Acute respiratory distress syndrome | Sufentanil: 20 µg/h  Midazolam: 5 mg/h | Spontaneous movement of the upper extremity, general weakness | PERRLA | Opens eyes spontaneously, moves head | Y | Y |
| P9 | 60 | Subarachnoid hemorrhage | none | No spontaneous movement | PERRLA | Opens eyes when talked to, Nods head | N | Y |
| P10 | 59 | Subarachnoid hemorrhage | Propofol: 150 mg/h  Sufentanil: 1,5 µg/h | No spontaneous movement | PERRLA | Opens eyes when talked to | Y | Y |
| P11 | 65 | Subarachnoid hemorrhage | Sufentanil: 37,5 µg/h  Midazolam: 37,5 mg/h  Ketamine: 375 mg/h | No spontaneous movement | PERRLA | No signs of activity | Y | - |
| P12 | 62 | Subarachnoid hemorrhage | Sufentanil: 17 µg/h  Midazolam: 17 mg/h | No spontaneous movement | PERRLA | No signs of activity | Y | - |
| P13 | 33 | Subarachnoid hemorrhage | Sufentanil: 12,75 µg/h  Midazolam: 7 mg/h | Spontaneous movement of the upper and lower extremity (left > right), but general weakness | PERRLA | - | Y | Y |
| P14 | 70 | Polytrauma | Midazolam: 12 mg/h | No spontaneous movement | PERRLA | No signs of activity | Y | - |
| P15 | 47 | Intracerebral hemorrhage | none | General weakness with rare spontaneous movement of the upper and lower extremities | PERRLA | Opens eyes on painful stimulus | N | Y |
| P16 | 72 | Subarachnoid hemorrhage | none | No spontaneous movement | Pupils anisocor (right > left), reactive to light | Opens eyes spontaneously | Y | Y |
| P17 | 63 | Intracerebral hemorrhage | none | General weakness (MRC 2-3/5), spontaneous movement of the upper and lower extremities; does not obey commands | PERRLA | Opens eyes spontaneously | Y | Y |
| P18 | 26 | Polytrauma | Sufentanil: 16 µg/h | General weakness, spontaneous movement of the upper and lower extremities; does not obey commands | PERRLA | Moves head | N | Y |

Y = Yes, N = No, - indicates that a conclusive clinical examination to decide on presence of intensive-care-unit-acquired weakness (ICU-AW) was not possible due to deep sedation or coma. PERRLA = Pupils equal round reactive to light and accommodation.
