# Supplementary material for: Facial nerve neurographies in intensive care unit-acquired weakness
Source: Neurol Res Pract. 2023 Sep 21;5:50. doi: 10.1186/s42466-023-00275-3 (PMC10512492; doi:10.1186/s42466-023-00275-3)
Supplement: Supplementary file 2 — Additional file 2. CMAPs in mV after distal stimulation of the peroneal, ulnar and right facial nerve and SNAPs after distal stimulation of the ulnar nerve. Course of the CMAPs after distal stimulation of the peroneal, ulnar and right facial nerve and course of the SNAPs after distal stimulation of the ulnar nerve during the study for each patient included, presented graphically as well as in a table. [file 42466_2023_275_MOESM2_ESM.docx]

Additional file 2

Pernoneal nerve stimulation

Ensemble plot demonstrating the course of the amplitudes of the compound motor action potentials (CMAPs) after distal stimulation of the peroneal nerve for each single patient included in the study. Y-axis = Amplitude in mV; D1 = day 1, D7 = day 7, D14 = day 14. P = Patient.

| P | Day 1 | Day 7 | Day 14 |
| --- | --- | --- | --- |
| 1 | 1 (0.6 mA) | 0.33 | 0.33 |
| 2 | 1 (1.9 mA) | 0.79 | 0.74 |
| 3 | 1 (0.7 mA) | 0.71 | 0.57 |
| 4 | 1 (1.8 mA) | 0.50 | 0.50 |
| 5 | 1 (3.1 mA) | 0.81 | 1.32 |
| 6 | 1 (0.2 mA) | 1.50 | 1.50 |
| 7 | 1 (2.8 mA) | 0.54 | 0.29 |
| 8 | 1 (0.1 mA) | 7.00 | 18.00 |
| 9 | 1 (3.5 mA) | 0.37 | 0.23 |
| 10 | 1 (3.4 mA) | 0.56 | 0.29 |
| 11 | 1 (4.2 mA) | 0.50 | 0.31 |
| 12 | 1 (4.4 mA) | 0.16 | 0.18 |
| 13 | 1 (2.0 mA) | 0.05 | 0.20 |
| 14 | 1 (2.2 mA) | 0.18 | 0.14 |
| 15 | 1 (1.2 mA) | 0.67 | 0.03 |
| 16 | 1 (3.7 mA) | 0.22 | 0.24 |
| 17 | 1 (1.5 mA) | 0.27 | 0.20 |
| 18 | 1 (3.5 mA) | 1.09 | 0.66 |
|  |  |  |  |
| M | 1 (2.1 mA) | 0.52 | 0.30 |

Table 1 shows the ratio of amplitudes of the measurements each compared to the baseline measurement at day 1 after distal stimulation of the peroneal nerve for each single patient included in the study. The actual amplitudes measured are given in brackets ± standard deviation for day 1.

Ulnar nerve stimulation

Ensemble plot demonstrating the course of the amplitudes of the compound motor action potentials (CMAPs) after distal stimulation of the ulnar nerve for each single patient included in the study. Y-axis = Amplitude in mV; D1 = day 1, D7 = day 7, D14 = day 14. P = Patient.

| P | Day 1 | Day 7 | Day 14 |
| --- | --- | --- | --- |
| 1 | 1 (3.0 mA) | 0.93 | 0.57 |
| 2 | 1 (5.8 mA) | 0.98 | 0.78 |
| 3 | 1 (2.5 mA) | 0.40 | 0.88 |
| 4 | 1 (2.8 mA) | 1.04 | 1.14 |
| 5 | 1 (2.9 mA) | 2.07 | 2.34 |
| 6 | 1 (3.1 mA) | 0.71 | 0.58 |
| 7 | 1 (5.4 mA) | 1.24 | 0.72 |
| 8 | 1 (3.6 mA) | 0.89 | 1.86 |
| 9 | 1 (4.9 mA) | 0.73 | 0.96 |
| 10 | 1 (5.6 mA) | 1.02 | 0.77 |
| 11 | 1 (3.3 mA) | 1.03 | 1.18 |
| 12 | 1 (4.6 mA) | 0.72 | 0.63 |
| 13 | 1 (8.2 mA) | 0.22 | 0.73 |
| 14 | 1 (3.5 mA) | 0.94 | 0.60 |
| 15 | 1 (7.4 mA) | 1.23 | 0.82 |
| 16 | 1 (3.6 mA) | 0.75 | 0.42 |
| 17 | 1 (4.9 mA) | 0.27 | 0.35 |
| 18 | 1 (5.5 mA) | 1.27 | 0.96 |
|  |  |  |  |
| M | 1 (4.1 mA) | 0.94 | 0.77 |

Table 2 shows the ratio of amplitudes of the measurements each compared to the baseline measurement at day 1 after distal stimulation of the peroneal nerve for each single patient included in the study. The actual amplitudes measured are given in brackets ± standard deviation for day 1.

Right facial nerve

Ensemble plot demonstrating the course of the amplitudes of the compound motor action potentials (CMAPs) after stimulation of the right facial nerve for each single patient included in the study. Y-axis = Amplitude in mV; D1 = day 1, D7 = day 7, D14 = day 14. P = Patient.

| P | Day 1 | Day 7 | Day 14 |
| --- | --- | --- | --- |
| 1 | 1 (1.6 mA) | 0.25 | 0.19 |
| 2 | 1 (1.2 mA) | 0.67 | 0.42 |
| 3 | 1 (0.6 mA) | 1.83 | 3.83 |
| 4 | 1 (0.7 mA) | 0.71 | 0.86 |
| 5 | 1 (0.7 mA) | 0.71 | 1.00 |
| 6 | 1 (0.7 mA) | 1.14 | 1.86 |
| 7 | 1 (1.5 mA) | 0.73 | 0.73 |
| 8 | 1 (0.3 mA) | 2.00 | 2.00 |
| 9 | 1 (0.5 mA) | 1.00 | 1.40 |
| 10 | 1 (1.2 mA) | 0.58 | 0.75 |
| 11 | 1 (0.7 mA) | 0.29 | 0.29 |
| 12 | 1 (1.0 mA) | 0.20 | 0.70 |
| 13 | 1 (0.7 mA) | 0.71 | 0.86 |
| 14 | 1 (0.2 mA) | 1.00 | 2.50 |
| 15 | 1 (2.2 mA) | 0.77 | 1.00 |
| 16 | 1 (0.4 mA) | 0.75 | 1.00 |
| 17 | 1 (0.8 mA) | 0.50 | 0.50 |
| 18 | 1 (2.6 mA) | 1.12 | 0.42 |
|  |  |  |  |
| M | 1 (0.7 mA) | 0.72 | 0.85 |

Table 3 shows the ratio of amplitudes of the measurements each compared to the baseline measurement at day 1 after stimulation of the right facial nerve for each single patient included in the study. The actual amplitudes measured are given in brackets ± standard deviation for day 1. Measurements for the left facial nerve were comparable and are not given here.

Amplitudes of the sensory ulnar nerve action potentials

Ensemble plot demonstrating the course of the amplitudes of the sensory nerve action potentails after stimulation of the ulnar nerve (mostly right) for all patients in whom this response could be elicited at each measurement. Y-axis = latency in ms; D1 = day 1. D7 = day 7. D14 = day 14. P = Patient.

| P | Day 1 | Day 7 | Day 14 |
| --- | --- | --- | --- |
| 1 | 1 (14 µV) | - | 1.07 |
| 2 | 1 (23 µV) | 1.04 | 0.43 |
| 3 | 1 (6 µV) | 1.00 | 1.50 |
| 4 | 1 µV | - | - |
| 5 | - | - | 18 µV |
| 6 | 6 µV | - | - |
| 7 | 1 (44 µV) | 0.30 | 0.34 |
| 8 | 1 (31 µV) | 0.13 | 0.97 |
| 9 | 1 (13 µV) | 1.08 | - |
| 10 | - | 17 µV | 3 µV |
| 11 | 2 µV | - |  |
| 12 | - | - | 34 µV |
| 13 | 1 (5 µV) | 4.20 | 2.20 |
| 14 | 1 (13 µV) | 1.85 | 0.62 |
| 15 | 1 (12 µV) | 1.17 | 2.33 |
| 16 | 1 (8 µV) | 0.88 | 0.63 |
| 17 | 1 (24 µV) | 0.75 | 0.46 |
| 18 | 1 (5 µV) | 1.20 | 5.00 |
|  |  |  |  |
| M | 1 | 1.23 | 1.41 |
| Med |  | 1.04 | 0.97 |

Table 4 shows the ratio of amplitudes of the sensory nerve action potentials (SNAP) after stimulation of one ulnar nerve each compared to the baseline measurement at day 1 for each single patient included in the study. The actual amplitudes measured are given in brackets ± standard deviation for day 1. Lower case numbers represent direct latencies of those cases in whom the SNAP could not be evoked at baseline. Due to the high variability of the data we calculated the median (med) aside of the mean (m).

R1-response of the orbicularis oculi reflex on the right

Ensemble plot demonstrating the course of the latencies of the ipsilateral R1-response after stimulation of the right supraorbital nerve for all eight patients in whom this response could be elicited at each measurement. Y-axis = latency in ms; D1 = day 1. D7 = day 7. D14 = day 14. P = Patient.

| P | Day 1 | Day 7 | Day 14 |
| --- | --- | --- | --- |
| 1 | 1 (12.1 ms) | - | - |
| 2 | 1 (11.4 ms) | 1.14 | 0.96 |
| 3 | 1 (10.3 ms) | 1.07 | 1.03 |
| 4 | 1 (11.6 ms) | 1.04 | 0.97 |
| 5 | 1 (10 ms) | 0.96 | 1.18 |
| 6 | - | - | 10.7 ms- |
| 7 | - | 11.6 ms | 12.8 ms |
| 8 | - | - | - |
| 9 | - | 12.2 ms | - |
| 10 | 1 (11.7 ms) | 1.09 | 1.01 |
| 11 | 1 (11.8 ms) | 1.04 | 1.03 |
| 12 | 1 (14.4 ms) | - | - |
| 13 | 1 (8.8 ms) | - | - |
| 14 | - | - | - |
| 15 | 1 (11.6 ms) | 1.07 | 1.12 |
| 16 | - | - | 11.3 ms |
| 17 | 1 (11.1 ms) | 0.93 | 0.96 |
| 18 | - | - | 9.8 ms |
|  |  |  |  |
| M | 1 | 1.04 | 1.03 |

Table 5 shows the ratio of latencies of the measurements each compared to the baseline measurement at day 1 after stimulation of the right supraorbital nerve in order to evoke an orbicularis oculi reflex for each single patient included in the study. The actual amplitudes measured are given in brackets ± standard deviation for day 1. Lower case numbers represent direct latencies of those cases in whom the reflex could not be evoked at baseline. Measurements for the left orbicularis oculi reflex were comparable and are not given here.
